# Is detection of intraperitoneal exfoliated tumor cells after surgical resection of rectal cancer a prognostic factor of survival?

**DOI:** 10.1186/s12885-017-3365-7

**Published:** 2017-06-07

**Authors:** Christian Arstad, Paulo Refinetti, Annette Torgunrud Kristensen, Karl-Erik Giercksky, Per Olaf Ekstrøm

**Affiliations:** 1 Department of Tumor Biology, Institute for Cancer Research, The Norwegian Radium Hospital, Oslo, Norway; 2 Chaire de Statistique Appliques, Section de Mathematiques, EPFL, Lausanne, Switzerland

**Keywords:** Rectal cancer, Prognostic factor, Survival, mtDNA mutations

## Abstract

**Background:**

The prognostic significance of free cancer cells detected in peritoneal fluid at the time of rectal surgery remains unclear. A substantial number of patients will develop metastatic disease even with successful local treatment. This prospective non-randomized study investigated the prognostic value of intraperitoneal free cancer cells harvested in peritoneal lavage after surgery for rectal cancer. Mutational hotspots in mitochondrial DNA were examined as potential molecular signatures to detect circulating intraperitoneal free cancer cells when present in primary tumor and in lavage.

**Methods:**

Point mutations in mitochondrial DNA amplifications were determined in primary tumors and corresponding exfoliated intraperitoneal free cancer cells in lavage from 191 patients with locally advanced rectal cancer scheduled for radical treatment. Mitochondrial DNA target sequences were amplified by polymerase chain reaction and base substitutions were detected by denaturant, cycling temperature capillary electrophoresis. Detection of intraperitoneal free cancer cells was correlated to survival***.***

**Results:**

Of 191patients analyzed, 138 (72%) were identified with somatic mitochondrial point mutations in rectal cancer tumors. From this fraction, 45 patients (33%) had positive lavage fluid with corresponding somatic mtDNA point mutations in lavage representing circulating intraperitoneal free cancer cells. There was no significant survival difference between patients identified with or without somatic mitochondrial DNA point mutations in the corresponding lavage.

**Conclusion:**

Somatic mitochondrial DNA point mutations identified in primary rectal tumors enable detection of circulating intraperitoneal free cancer cells in lavage fluid.

Intraperitoneal free cancer cells harvested from lavage immediately after surgery for rectal cancer does not represent an independent prognostic factor on survival.

## Background

Rectal cancer is frequent in both genders. In 2014 the incidence in Norway was 1365 new cases in a population of 5.05 million citizens. [1]. Randomized studies have demonstrated increased long-term survival in rectal cancer patients receiving chemoradiotherapy in combination with the surgical technique of total mesorectal excision (TME [2]. A substantial number of patients develops local recurrence and/or metastatic disease despite successful local treatment [3, 4]. Detection of intraperitoneal free cancer cells (IPCC) is considered an important prognostic tool in ovarian and gastric cancers [5–11]. In colorectal cancer IPCC with proliferate or metastatic potential may originate from the primary tumor and can possibly be detected by peritoneal lavage [12]. Detection of IPCC during surgery may be of relevance to improve staging and eventually characterize patients who may benefit from aggressive multimodal treatment [12]. However, the clinical significance of detecting IPCC in colorectal cancer is still debated [12–15]. Different yield rates (2, 2–31%) of positive IPCC detection reported in colorectal cancer may be an explanation [12]. Lavage analysis techniques (Cytology, PCR, Immunocytochemistry) used to identify IPCC vary with an average of 13%, as calculated from 11 studies involving more than 100 patients, reviewed by Passot et al. [12]. The use of more sensitive methods to detect IPCC in studies with a large number of patients could determine if IPCC is of clinical relevance.

Somatic mitochondrial DNA (mtDNA) mutations are observed in large fractions of tumors *(Manuscript in press).* Utilizing somatic mtDNA mutations in the primary tumor can be used as marker to detect IPCC. The axiom is that detection of the same mtDNA mutation in the primary tumor and in the lavage fluid represents IPCC. This study analyses primary tumors and lavage from rectal cancer patients only. The aim of this study was to examine the prognostic impact of IPCC if detected after resection of locally advanced primary or local recurrent rectal cancer.

## Methods

### Study population

The Norwegian Radium Hospital is a tertiary referral center for locally advanced primary and recurrent rectal cancer. Hundred and ninety-one patients with either locally advanced or local recurrent rectal cancer (TNM stage II and III) receiving CRT and subsequent surgery (TME) at the Norwegian Radium Hospital in the period 2000–2006. All patients were of Caucasian origin. Patient’s age ranged from 30 to 87 years with a median age of 66 (55% men, 45% women). Summary of patient staging can be found in the supplementary material. Surgery was performed a median 56 days after completion of radiotherapy.

### Follow-up

Initially these patients were included in a pilot study where *KRAS* mutations in exon 1 identified in primary rectal tumors and lavage was reported to be an independent poor prognostic factor for overall survival. [16]. The data is reexamined with extended observation time, and consequently no censored data. The results debate previous findings, of which could have been biased by a large fraction of censored data.

### Tumor samples

Rectal tumor tissue samples harvested during surgery were submerged in an appropriate volume of RNAlater (Qiagen, Valencia, California, USA) After RNAlater removal the samples were frozen at −80 °C until DNA extraction. QIAamp DNA Kit (Qiagen, Valencia, California, USA) was used for the DNA extraction, following the manufacturer’s instructions. After resection of the tumor, the pelvis was washed with sterile water 200–600 ml (discarded) subsequently followed by 200–600 ml saline water. Two specimens (lavage A and B) of 50 ml lavage fluid were aspirated to centrifuge tubes. Cells were harvested by centrifugation at 1200G for 10 min followed by removal of the supernatant. The cell pellets were frozen at −20 °C until DNA extractions were performed. QIAamp DNA Kit (Qiagen, Valencia, California, USA) was used for the DNA extraction, following the manufacturer’s instructions.

### First round PCR

Amplification of mtDNA was performed using a two step nested PCR protocol to avoid amplification of homologous regions in the nuclear DNA. A set of 5 specific mitochondrial primers were used in the first round PCR to amplify the base pair regions: 15,924–201, 16,521–880, 6917–7671, 10,852–11,566, 15,169–15,993, according to the reference mitochondrial build NC_012920.1. Primer sequences are displayed in Table 1. The PCR reaction mixture contained 0.1 μl of extracted DNA, 0.8 mM dNTPs (0.2 mM of each dNTP) (VWR, Oslo, Norway), 1X Thermopol Buffer, 2 mM MgSO_4_, 0.075unit Taq/μl, 0.15 μM of each forward, reverse and fluorescently labeled primer (Integrated DNA Technologies, Leuven, Belgium) and total reaction volume of 10 μl. The temperature cycling was performed in a Eppendorf Mastercycler ep gradient S (Eppendorf, Hamburg, Germany) with an initial denaturation 94 °C for 240 s followed by cycling 38 times under the following conditions, denaturation at 94 °C for 15 s, annealing for 40 s with temperature given in Table 1 and elongation at 72 °C for 150 s.

**Table 1:** Primers used in specific mtDNA amplification

**#**	Start(bp)	End(bp)	Length(bp)	“Forward” primer (5′–3′)	“Reverse” primer (5′–3′)	Annealing temperature (°C)
23	15,924	201	846	^a^AACCGGAGACGAAAACCTTTTTC	^a^CTTTAGTAGGTATGTTCGCCTGT	51
1	16,521	880	928	^a^CCATAAAGCCTAAATAGCCCACA	^a^CCAACCCTGGGGTTAGTATAGCT	54
10	6917	7671	754	^a^TGCTCTGAGCCCTAGGATTCATC	^a^TGAGGGCGTGATCATGAAAGGTG	55.5
16	10,852	11,566	714	^a^GCCTAATTATTAGCATCATCCCC	^a^ATGCCTCATAGGGATAGTACAAG	51
22	15,169	15,993	824	^a^GAGGGGCCACAGTAATTACAAAC	^a^TGGGTGCTAATGGTGGAGTTAAA	51

^a^=tail sequence (CGCCCGCCGCGCCCCGCG)

### Capillary electrophoresis

First round amplification products were verified by capillary electrophoresis in MegaBACE 1000 DNA Analysis System (GE Healthcare Life Sciences, Pittsburgh, PA, USA). Samples were loaded into the capillaries from 96-well plates by electro kinetic injection at 161 V/cm for 20 s. The temperature of the capillary chamber was set to 27 °C and electrophoresis was carried out at a constant field of 145 V/cm.

### Second round PCR

Templates for second round PCR were 0.8 μl of a 1:200 dilution (first round PCR in H_2_O). The templates were dispensed into 96-wells plates with a syringe dispenser (Hydra 96, Robbins Scientific, USA). To each well 10 μl reaction mixture was added. The components had a final concentration; 1xThermopol Reaction Buffer with 2 mM MgS0_4_, 0.3 μM primers without GC clamp, 0.15 μM 1/2GC-tailed primer, 0.15 μM, 6-Carboxyfluorescein-GC-clamp, 500 μM dNTP, 100 μg Bovine Serum Albumine (Sigma-Aldrich, Oslo, Norway) and 0.75 U Cloned Pfu DNA polymerase. Plates were sealed with two strips of electrical tape (Clas Ohlson, Oslo, Norway). The temperature cycling was repeated 30 times; 94 °C for 15 s, annealing temperature held for 30 s and extension at 72 °C for 60 s.

Primer sequences are displayed in Table 2.

**Table 2:** Primers used in second round PCR amplification

#	Start (bp)	End (bp)	Template, fragment # from # from	“Forwards” primer (5′ - 3′)	“Reverse” primer (5′ - 3′)	Annealing temperature (°C)
1	16,569	25	1	^a^TGCATGGAGAGCTCCCGTGAGTGG	CCCCTTAAATAAGACATCACGAT	52
2	42	126	1	^a^ATTAACCACTCACGGGAGCTCTC	AGGATGAGGCAGGAATCAAAGAC	55
4	131	181	1	^a^CACCCTATGTCGCAGTATCTGTC	CACACTTTAGTAAGTATGTTCGC	55
6	483	513	1	^a^GGGGTTAGCAGCGGTGTGTGTGTG	TCCCACTCCCATACTACTAATCT	55
7	530	633	1	^a^TACCCAGCACACACACACCGCTG	CAAACCTATTTGTTTATGGGGTGA	55
8	673	705	1	^a^TTAGAGGGTGAACTCACTGGAAC	GGTTTGGTCCTAGCCTTTCTATT	58
82	7031	7134	10	^a^ACGACACGTACTACGTTGTAGCC	AATATGATAGTGAAATGGATTTT	52
84	7340	7416	10	^a^CTTTCTTCCCACAACACTTT CT C	TCTCAAATCATGAAAATTATTAAT	55
125	11,029	11,086	16	^a^TTAGGAGGGGGGTTGTTAGGGGGT	CATCCCTCTACTATTTTTTAACC	58
127	11,193	11,243	16	^a^ACCAGCCAGAACGCCTGAACGCA	GGTGTTGTGAGTGTAAATTAGT G	55
128	11,283	11,311	16	^a^TGTGCCTGCGTTCAGGCGTTCTGG	TAATCATATTTTATATCTTCTTC	60
130	11,437	11,492	16	^a^TTGACCCAGCGATGGGGGCTTCGA	GAGCCAACAACTTAATATGACTA	55
176	15,201	15,257	22	^a^AGAATCGTGTGAGGGTGGGACTGT	AGTAATTACAAACTTACTATCCG	60
177	15,274	15,377	22	^a^AGTAGACAGTCCCACCCTCACAC	GGTGATTTTATCGGAATGGGAGG	60
178	15,394	15,448	22	^a^CTAGGAATCACCTCCCATTCCGA	TAATGTCATTAAGGAGAGAAGGAA	55
181	15,761	15,864	22	^a^ACCTCCTCATTCTAACCTGAATC	CAGGCCCATTTGAGTATTTTGTTT	55
184	16,080	16,130	23	^a^CAAGTATTGACTCACCCATCAAC	ACAGGTGGTCAAGTATTTATGGTA	57
187	16,263	16,366	23	^a^AACTGCAACTCCAAAGCCACCCC	CCCTATCTGAGGGGGGTCATCCAT	58

^a^=tail sequence (CCCGCCGCCCCCGCCCGGG)

GC-Clamp = (6FAM-GCGCCCGCCGCGCCCCGCGCCCGTCC CGCCGCCCCCGCCCGGG)

### Cycling temperature capillary electrophoresis

6-Carboxyfluorescein labeled PCR products were analyzed in a 96-capillary DNA analyzer MegaBACE 1000. The instrument was modified as previously described to allow for elevated temperature cycling [17, 18]. The cycling temperature was based on the theoretical melting temperature, for each fragment, calculated by Poland’s algorithm in the implementation described by Steger [19]. The separation temperature proposed by the algorithms was adjusted based on the urea concentration in the matrix. The cycling temperature was programmed in the macro. Ini file used by the Instrument Control Manager (ICM) software package (GE Healthcare Life Sciences, Pittsburgh, PA, USA). The injection and running electric fields were as given for the first round amplicons.

### Follow up & statistics

Patient’s survival data for 60 months was obtained from the Norwegian National Health Register. Data analysis was performed using SPSS 23.0 for Windows (SPSS Inc. Chicago IL, USA). The log-rank test assessed the differences in survival between groups and cumulative survival was demonstrated using the Kaplan-Meier plot. *P*-values of less than 0,05 were considered statistically significant.

Area under the curves was measured by use of AcqKnowledge ® 4.4.1 Software & MP150/MP36R in electropherograms displaying low mutant fractions.

## Results

**Fig. 1:**
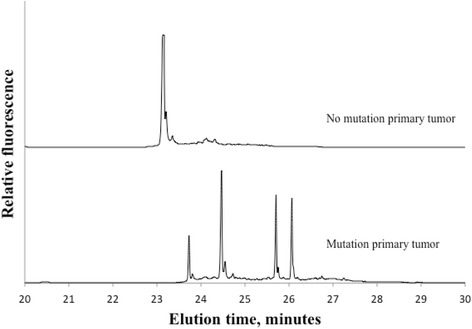
Electropherogram of primary rectal tumor without mtDNA mutations (top) and primary rectal tumor with mtDNA mutations (lower)

**Fig. 2:**
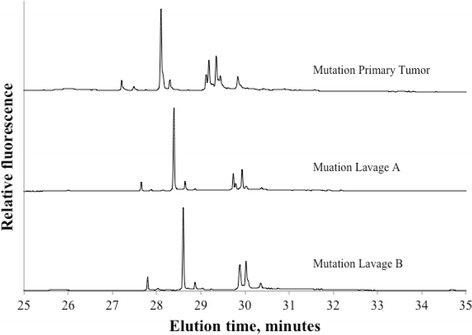
Electropherograms of mutant positive primary rectal tumor with mtDNA mutations and in corresponding lavage A & B

**Fig. 3:**
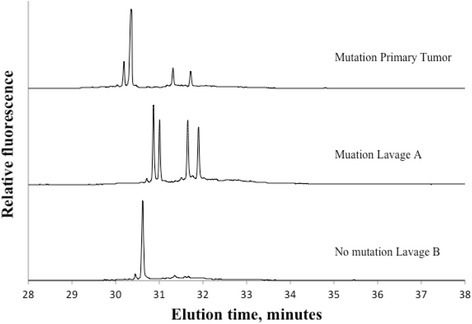
Electropherograms of primary rectal tumor with mtDNA mutations and mtDNA mutations in lavage A, but not in lavage B

**Fig. 4:**
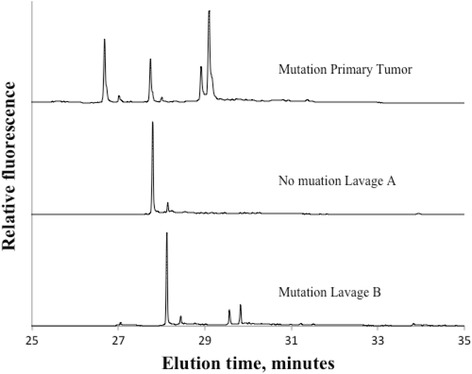
Electropherograms of primary rectal tumor with mtDNA mutations and without mtDNA mutations in lavage A, but with mutation in lavage B

Of 191 locally advanced or local recurrent rectal tumors 72% (138/191) were found positive for at least one mtDNA mutation. Figure 1 displays two representative electropherograms of non mutated and mutated samples. Lavage fluids from these 138 patients were subsequently analyzed for corresponding mtDNA mutations. Forty-five of the lavages were identified with equivalent mtDNA mutations as in the primary tumor, although in different fractions. Figure 2 demonstrates mutations in tumor and in both collections of lavage. Figures 3 and 4 show positive mutant marker only in one of the lavage samples.

**Fig. 5:**
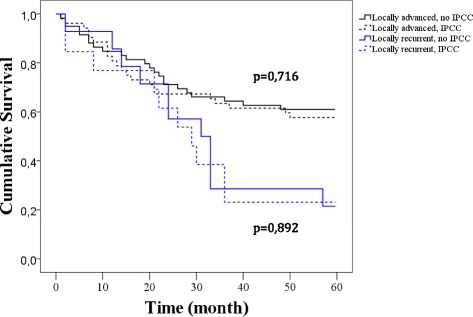
Kaplan-Meier plot. Survival analysis, calculated from patients with locally advanced and locally recurrent rectal cancer, having positive IPCC or negative IPCC. No censored data

By visual inspection of all lavage electropherograms, the mitochondrial mutant fraction in the sample was measured. Based on these data lower limit of detection was calculated to be1%. For survival analysis, a Kaplan-Meier plot was prepared from patients with locally advanced and local recurrent rectal-cancer with positive and negative IPCC in the lavage fluid (Fig. 5).

Observation time was 60 months for all patients. The log-rank test assessed no significant difference in survival between the positive and negative IPCC for the two groups (*p* = 0,716 and *p* = 0,892).

**Fig. 6:**
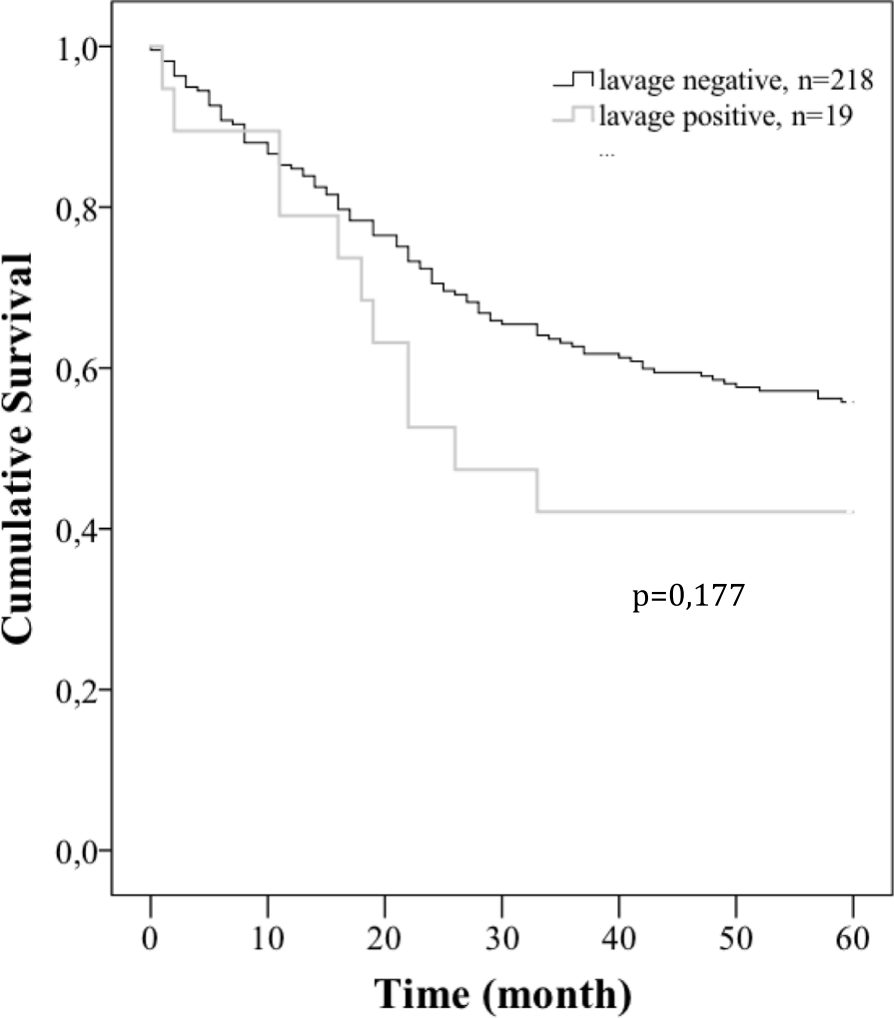
Kaplan-Meier plot. Survival analysis, calculated from patients with KRAS mutations positive vs. KRAS mutations negative in lavage fluid. No censored data (*P* = 0,177)

Initially these patients were included in a pilot study where *KRAS* mutations in exon 1 identified in primary rectal tumors and lavage was reported to be an independent poor prognostic factor for overall survival [16]. Reevaluation of the data with extended time of observation did not validate this assertion. The log-rank test with uncensored data (60 months) gave no significant difference (*p* = 0,177) in survival between positive and negative *KRAS* mutations in lavage fluid (Fig. 6). When comparing the current patient material with the previous published population [16] no difference in TNM staging was observed (Additional file).

## Discussion

This prospective non-randomized study comprises 191 patients with locally advanced rectal cancer receiving CRT followed by radical surgery.Local recurrence following CRT and TME cannot be explained only by incomplete surgery, vascular or lymphatic invasion [20]. Pelvic IPCC with proliferate or metastatic potential may originate from the primary tumor transmitted either prior to or during surgical procedure and could represent a source of recurrence. [12]. The survival impact of IPCC in lavage harvested after surgery for rectal cancer was investigated. The mtDNA of primary rectal tumors were scanned for mutations. The fragments used are those covering mtDNA hot spots.The mtDNA hot spots were determined by CTCE when scanning 76% of the genome in 94 tumors of different origin *(Manuscript in press)*. Observing the same mtDNA mutations in primary tumor and lavage supports the presence of IPCC and allowed a simplified experimental protocol. The study population constitutes 138 patients with a positive mtDNA tumor marker and from these a fraction of 33% (45/138) was identified with a corresponding marker in lavage. By detecting the same mtDNA mutation in primary tumor and lavage, was hypothesized to confirm the existence of IPCC. The lavages from 53 tumors without mtDNA mutations were not further analyzed. A yield rate of 33% was considered sufficient to detect a possible effect on survival [12]. The steps in IPCC detection include collection procedures and sample treatment, cell separation protocols and chosen biomarkers. Essential to all these methods is the recognition of markers assumed to exclusively represent tumor cells and suspending normal tissue in the examined samples. Due to non-specific labeling, cytology based methods and immunohistochemistry in IPCC detection reports conflicting results [21–23]. Whole genome sequencing is time-consuming, expensive, and impractical for routine analysis and is still left with challenges [24–28]. The combination of a two staged PCR followed by CTCE represents a quantitative, fast and inexpensive method with sufficient through-put to detect mutations in human mtDNA [29]. The standard procedure of peritoneal lavage at the time of study combines sterilized water followed by saline water performed after surgery. Assuming that exfoliated tumor cells will be compromised during exposure to lavage with sterilized water, the tumor cell viability was not tested. The lavage fluid includes circulating DNA generated from bleeding, lymphatic drainage, and tissue damage during surgery and IPCC if present. The detection limit for the method used is in the order of 1% [29]. With a detection limit at 1% (sensitivity), the 67% of lavage fluids without correlating mtDNA mutations, can be considered IPCC deficient. False negative signals may occur as a result of lavage contamination with white blood cells. One ml of blood contains an average of 5 × 10^6^ white blood cells. Consequently inevitable surgical bleeding may dilute the IPCC signal below detection limit. The probability of a sub population of cells to acquire mutations as observed in the primary tumor is less than 1:1000 (unpublished data), thus justifying the hypothesis that the IPCC signal is derived from the primary tumor. Observing coexisting mtDNA mutations in primary tumor and lavage identifies one tumor lineage. However, all possible tumor lineages are not necessarily identified. When analyzing tumor and lavage, four possible analytical outcomes are to be expected. First, detecting positive marker in tumor and lavage (Figs. 2, 3 and 4). This observation is the only combination confirming the presence of IPCC. Second and third, detecting positive marker in tumor or lavage, while the respectively lavage and tumor are negative for the marker. These outcomes do not exclude IPCC if the tumor lineage is devoid of the mtDNA mutation examined or below detection limit of the assay. Fourth, when tumor and lavage does not contain the marker, possible IPCC cannot be excluded. Consequently, 75% of the assay information is non-informative concerning IPCC status. Hence, a prerequisite for analyzing lavage samples was positive mtDNA mutations detected in primary tumors. The frequency of primary rectal tumors with mtDNA mutation in hot-spot fragments was found to be 72%. From these, 33% had the same mtDNA mutations in the primary tumor and lavage, and was interpreted to represent IPCC. The ambition of pelvic exposure to sterilized water after surgery is to compromise the viability of possible remnants with proliferate or metastatic potential. The mtDNA molecular signal does not discriminate IPCC from their residues. A yield rate of 33% of IPCC detected exceeds upper limits of previous reports [12]. The survival analysis, comparing patients with positive or negative IPCC in lavage fluid disclosed no significant difference on survival (Fig. 5). This is in contrast with former report on the same patient material using *KRAS* mutations as IPCC marker [16]. However, at the time of the previous study, a large fraction of the population was censored, as the observation time was not completed. In accordance with the authors of the previous article [16], the data was re-evaluated, with all patients having their observation period of 60 month complete, and thus no censored data. This reevaluation did not confirm the original observation. Detection of KRAS mutation in lavage fluid have no statistically significant effect on survival (Fig. 6)*.* This observation is in concordance with the lead author of the original research article (co-writing this paper) and will be conveyed to the publisher (***BMC Cancer***).

## Conclusion

Mutations in mtDNA can be detected in locally advanced or local recurrent rectal tumors and followed in exfoliated tumor cells with a detection limit of 1%.

The impact of finding the coexisting mutation in primary tumor and lavage disclosed no prognostic significance on survival.

KRAS mutations identified in locally advanced or local recurrent rectal tumors and lavage formerly labeled to be an independent poor prognostic factor on overall survival was not validated.

## Abbreviations

CRT: Chemo radiotherapy
CTCE: Cycling temperature capillary electrophoresis
IPCC: Intraperitoneal free cancer cells
MtDNA: Mitochondrial DNA
PCR: Polymerase chain reaction
TME: Total mesorectal excision
